# Integration of a Passive Exoskeleton and a Robotic Supernumerary Finger for Grasping Compensation in Chronic Stroke Patients: The SoftPro Wearable System

**DOI:** 10.3389/frobt.2021.661354

**Published:** 2021-06-10

**Authors:** Gionata Salvietti, Leonardo Franco, Martin Tschiersky, Gerjan Wolterink, Matteo Bianchi, Antonio Bicchi, Federica Barontini, Manuel Catalano, Giorgio Grioli, Mattia Poggiani, Simone Rossi, Domenico Prattichizzo

**Affiliations:** ^1^ Siena Robotics and Systems Laboratory Group, Department of Information Engineering and Mathematical Science, University of Siena, Siena, Italy; ^2^ Chair of Precision Engineering, Department of Engineering Technology, University of Twente, Enschede, Netherlands; ^3^ Biomedical Signals and Systems (BSS) and Robotics and Mechatronics (RAM) Group, Department of Electrical Engineering, Mathematics and Computer Science, University of Twente, Enschede, Netherlands; ^4^ Research Centre “E. Piaggio” and Department of Information Engineering, University of Pisa, Pisa, Italy; ^5^ Soft Robotics for Human Cooperation and Rehabilitation, Istituto Italiano di Tecnologia, Genova, Italy; ^6^ Brain Investigation and Neuromodulation Lab, Department of Medicine, Surgery and Neuroscience, University of Siena, Siena, Italy

**Keywords:** assistive robotics, wearable robotics, exoskeletons, human-robot interfaces, upper-limb impairments

## Abstract

Upper-limb impairments are all-pervasive in Activities of Daily Living (ADLs). As a consequence, people affected by a loss of arm function must endure severe limitations. To compensate for the lack of a functional arm and hand, we developed a wearable system that combines different assistive technologies including sensing, haptics, orthotics and robotics. The result is a device that helps lifting the forearm by means of a passive exoskeleton and improves the grasping ability of the impaired hand by employing a wearable robotic supernumerary finger. A pilot study involving 3 patients, which was conducted to test the capability of the device to assist in performing ADLs, confirmed its usefulness and serves as a first step in the investigation of novel paradigms for robotic assistance.

## 1 Introduction

Stroke is a main source of long-term impairments of the upper limb (Go et al., 2014). For affected people, the restoration of hand and arm function is particularly important to execute Activities of Daily Living (ADLs). Robotic aids represent promising tools for the recovery of a post-stroke paretic upper limb. A few devices have been created to provide supervised intensive rehabilitation training to patients with minor to severe motor disabilities after neurologic injury (Kwakkel et al., 2007; Lo et al., 2010). The use of robotic devices in rehabilitation and assistance can enable intense, engaging and targeted treatment of the debilitated arm, and can serve as a reliable method for observing patient advancement (Chiri et al., 2012). A comprehensive review on robot-assisted therapy for hand treatment can be found in Lum et al. (2012). In Heo et al. (2012), the authors presented a broad survey on hand exoskeleton innovations for rehabilitation and assistance. However, most of these devices have low wearability, and are intended to restore functional motions during the first months after stroke, when typically plastic changes of the central nervous system occur. Hardly any assistive devices are intended to effectively restore hand functions for patients in a chronic state.

In the context of the European SoftPro^1^ project, a consortium of universities and companies have investigated novel solutions for assistive robotic tools to be used at home by chronic stroke patients (Piazza et al., 2018). As pointed out by Amirabdollahian et al. (2014), it is important that exoskeletons increase the frequency and accessibility of physical therapy. Traditional exoskeleton approaches include specific user interfaces for training (Ambrosini et al., 2017; Zeiaee et al., 2017), and are barely portable (Housman et al., 2007; Nef et al., 2009; Frisoli et al., 2011). We proposed the use of wearable robots as assistive tools for recovering grasping capabilities in patients with paretic arms. A prime example of such an assistive wearable robot is the Robotic Sixth Finger (Salvietti et al., 2017), a soft robotic finger worn at the wrist of the paretic limb. This robotic finger can be used to achieve a stable grasp by combining its flexion capability with the adjacent paretic limb that acts as a palm to stabilize the grip. The Robotic Sixth Finger can be used by patients that maintain the ability to move the forearm against gravity after stroke, allowing them to perform bimanual tasks. In the SoftPro project, we have studied the combination of the Sixth Finger with a passive and lightweight elbow exoskeleton called Assistive Elbow Orthosis, an instrumented cap which is a human-robot interface called the e-Cap, and a force feedback device called the CUFF. Combining these devices we aim to expand the possibilities of using the Sixth Finger. With respect to state-of-the-art assistive devices, the design proposed in this work is very lightweight and would enable chronic stroke patients to perform ADLs which require a bimanual grasp.

Another important aspect that has to be considered when designing assistive tools is the user interface. Such an interface must be intuitive, also considering possible neurological deficits after stroke, while being highly reliable and robust. We achieved these requirements by designing an sEMG interface embedded in a cap, called e-Cap, for the Sixth Finger control and a wearable haptic interface, called CUFF, for force feedback from the wearable robotic finger.

**FIGURE 1:**
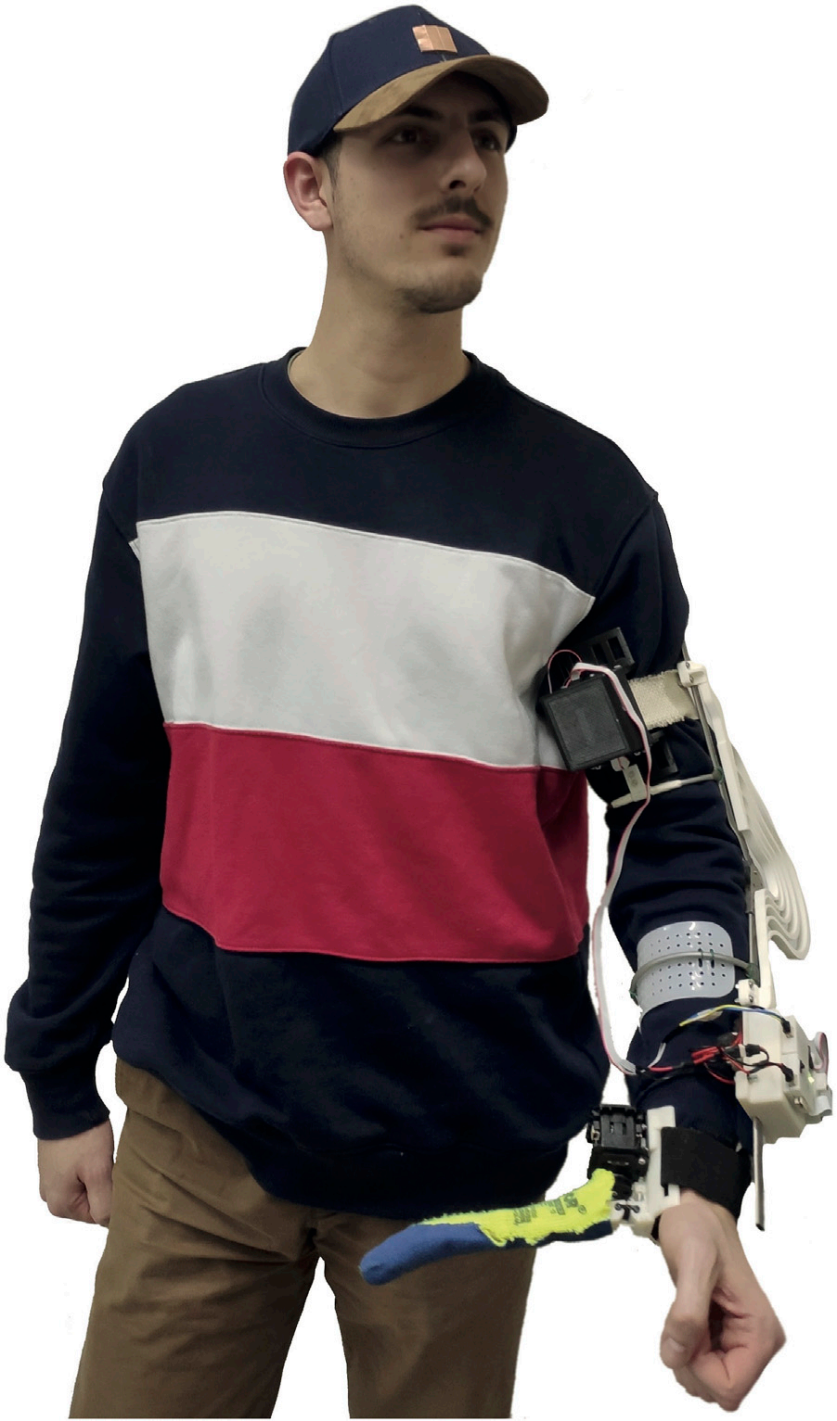
The SoftPro wearable system worn by a healthy subject.

In this paper, we report how we have integrated these components into a single easy-to-wear device, see Figure 1, as well as the results of a pilot study involving three stroke patients.

The rest of the paper is organized as follows: in section 2 all devices that are involved in the integration are presented and described in detail. In section 3, we explain how the integrated system works and how we modified each of the components to match the technical requirements. In sections 4 and 5 we report on the pilot study that involved three patients and show the preliminary results, respectively. In the last two sections, 6 and 7, a discussion is presented and conclusions are outlined.

## 2 System Components

The rationale for this device integration is to restore upper-limb function that a person looses due to stroke. All technologies in this integration are focused on restoring the functionality of the impaired upper limb. The intended use of the system mostly focuses on bimanual ADLs, for instance, opening a bottle. Analyzing this apparently simple task in more detail, we identify two concurrent sub-tasks: stabilizing the bottle and unscrewing the bottle cap. The proposed system can be used to assist the paretic arm to grasp and thus stabilize the bottle, whereas the healthy arm is used to perform the more dexterous sub-task of unscrewing the bottle cap.

The individual components of the proposed system have been developed by different research laboratories to be used independently from each other. The only devices that have been developed contextually and were previously integrated are the e-Cap and the Sixth Finger, both developed by the University of Siena (US). The aim of the Sixth Finger is to restore the ability to grasp objects with a paretic hand, allowing bimanual tasks. The e-Cap was developed to allow intuitive operability of the Sixth Finger. Before the development of this human-robot interface the Sixth Finger was used by means of a ring with buttons on the healthy hand of the stroke patient. The device was developed since stroke patients reported a lack of usefulness when occupying the healthy hand by operating the Sixth Finger grasping action at the impaired hand. To solve this issue, University of Siena developed the e-Cap, a human-robot interface embedded in a regular cap to ease wearing. The device is designed to recognize the movement of the eyebrows through real-time sEMG measurement of the frontalis muscle (Hussain et al., 2016; Salvietti et al., 2017). In Franco et al. (2019), the authors have shown that a vibrotactile feedback at the occipital area of the head can be used as an acknowledgement of the correct processing of the sEMG signal improving the usability of the interface. In this way, whenever the user moves the eyebrows to control the Sixth Finger, the e-Cap informs the user with a short vibration burst that the Sixth Finger is about to close or to open.

The integration presented in this paper extended the feedback feature, mapping the motor current of the Sixth Finger, an estimate of the force exerted onto the grasped object, to the CUFF (Casini et al., 2015). The CUFF interface has been developed by the University of Pisa (UP) in collaboration with the Istituto Italiano di Tecnologia (IIT). It can render a real-time force feedback by squeezing the arm through an actuated fabric belt. Therefore, in addition to a discrete vibration feedback, the integrated device also features a continuous force feedback. Furthermore, the two haptic devices use different modalities, as the one for input acknowledgement is vibrotactile, while the one for force feedback is based on skin stretch.

The Sixth Finger and the CUFF are worn on the paretic arm together with the necessary batteries for power supply. This added weight can further reduce the mobility of the limb in some patients. To address this issue, all devices, except for the e-Cap, were mounted onto the Assistive Elbow Orthosis (Tschiersky et al., 2019), a passive gravity-balancing device developed by the University of Twente (UT), featuring a rigid-link arm brace (Plettenburg, 2007) and a 3D-printed spring which is designed to provide weight compensation to the forearm of the affected limb. By tuning the dimensions of the spring, the amount of weight compensation can be adjusted to include the weight of the added devices and facilitate elbow flexion.

In the rest of this section, we will briefly present all devices that were embedded in the wearable robotic system: the Robotic Sixth Finger, the e-Cap, the CUFF, and the Assistive Elbow Orthosis.

### 2.1 Robotic Sixth Finger

The Robotic Sixth Finger is a wearable supernumerary robotic finger. It acts as a functional replacement of the thumb, and has been demonstrated to compensate missing grasping capabilities in stroke subjects (Hussain et al., 2016; Hussain et al., 2017; Salvietti et al., 2017).

**FIGURE 2:**
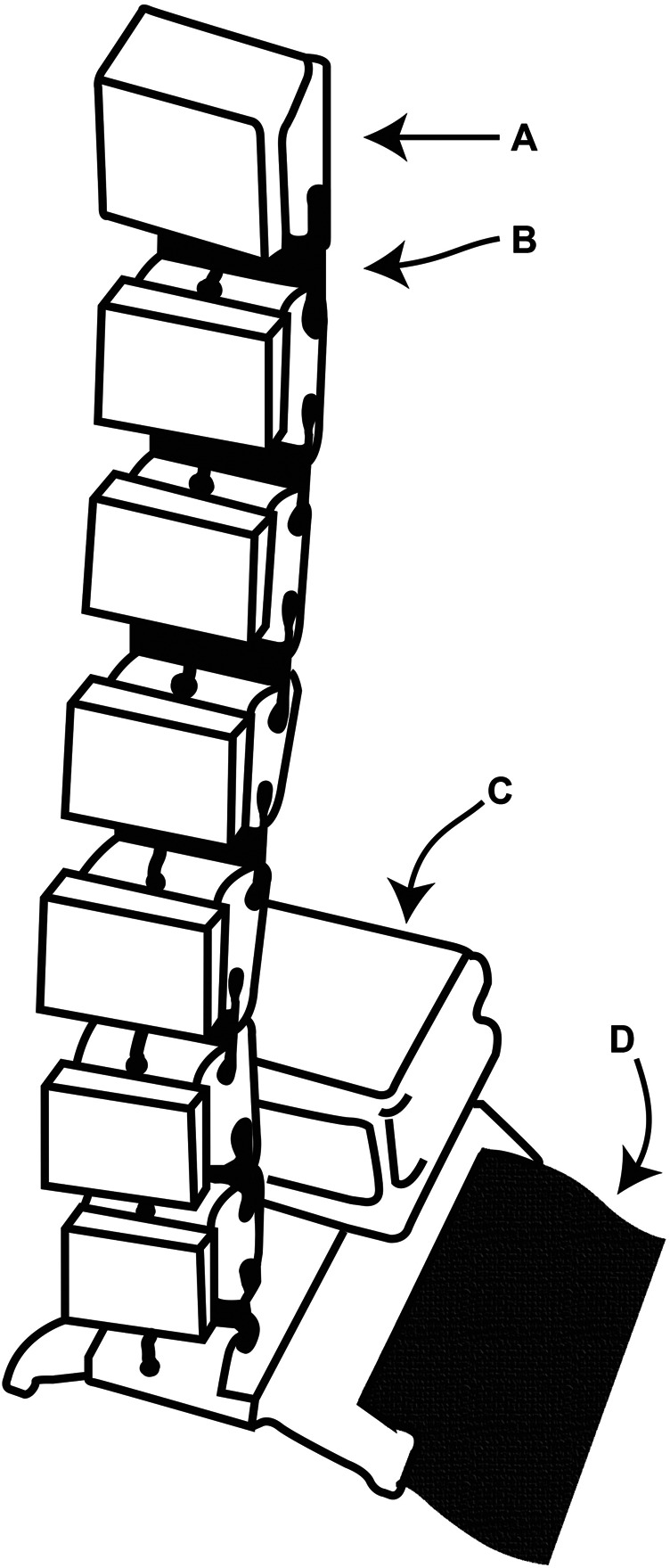
The Robotic sixth finger. **(A)** 3D-printed rigid link. **(B)** 3D-printed flexible link. **(C)** Dynamixel MX-28 servomotor. **(D)** Velcro strip mounted on the rigid 3D-printed base.

As shown in Figure 2, the Robotic Sixth Finger is a modular underactuated robotic finger, composed of rigid and flexible links which are driven by a motor *via* a tendon.

In the SoftPro Wearable System, the Robotic Sixth Finger enables the stroke patient to grasp objects in combination with the impaired limb. The device has been completely redesigned to improve wearability, long operation time and to cover a broad range of graspable objects. To this end, a Dynamixel MX-28 motor and 32,000 mAh Li-Po Batteries are used. Stroke patients experienced difficulties in lifting objects with the added burden of the Robotic Sixth Finger weight. To solve this issue, the 3D-printed base of the device has been modified to be rigidly attached to the Assistive Elbow Orthosis, that will be described comprehensively in Section 2.4. This modification mitigates the problem of lifting the impaired forearm, since the weights of devices are offloaded to the exoskeleton.

Regarding the software, the code is stored and runs on the Robotic Finger microcontroller, a Teensy 3.2. The Finger’s movements are coded in a finite state machine, described in Hussain et al. (2016). This part of the code is unchanged. We chose to use the Sixth Finger microcontroller to be the master device, since it was easy to program, and used the CUFF as a slave device. We will describe the hardware and software modifications that allow these devices to communicate in Section 3.

### 2.2 e-Cap

**FIGURE 3:**
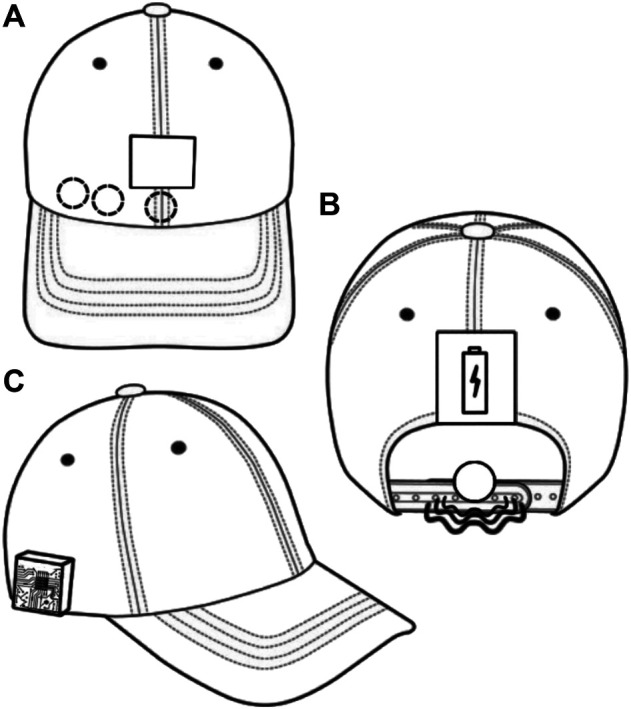
The e-Cap. **(A)** Front view (electrode positions indicated by dashed circles, touch interface by solid rectangle). **(B)** Rear view (Vibromotor indicated by solid circle and waves, battery by solid rectangle). **(C)** Side view (showing the electronics box).

**FIGURE 4:**
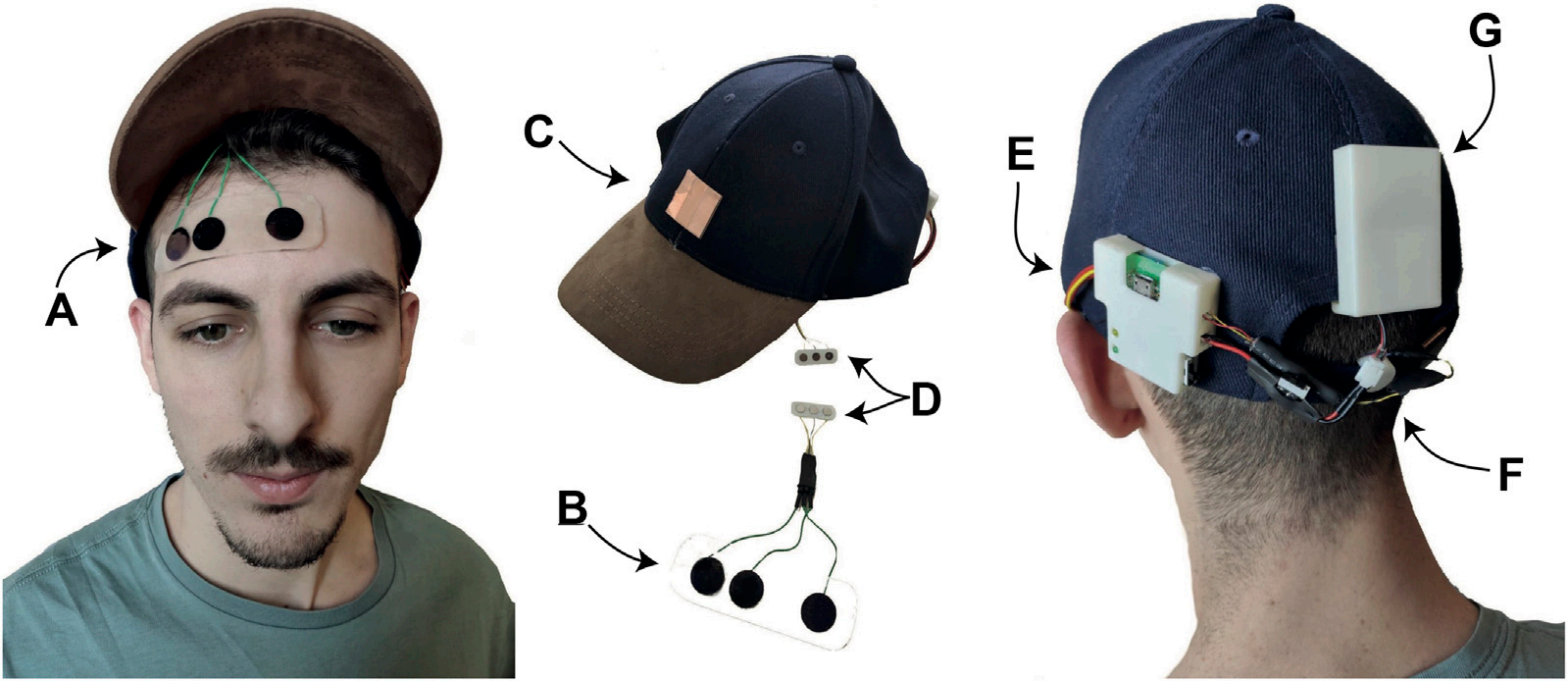
The novel e-Cap. **(A,B)** 3D-printed electrodes by UT combined with the support for easy fixation at the forehead. **(C)** Touch button for calibration procedure. **(D)** Magnetic connection for easy connection of the electrodes to the cap. **(E)** Control board. **(G)** Power supply. **(F)** Vibrotactile motor for haptic feedback.

The e-Cap has been redesigned to be integrated into the novel system. As shown in Figures 3, 4, the e-Cap consists of an sEMG acquisition chain composed of dry electrodes that will be described in Section 2.3, a commercial instrumentation amplifier and a Teensy 3.2 microcontroller to sample the analog signal. The e-Cap electronic board is equipped with a Bluetooth antenna (RN-42) which is used to stream data to the Sixth Finger. The on-board microcontroller samples the sEMG signal, processes it to extract the command for the Sixth Finger, conveys it to the Sixth Finger and generates an acknowledgement haptic feedback that is sent in real-time to a vibromotor placed at the back of the head. Finally, a 3D-printed box for the battery is located at the back of the cap.

Being an sEMG-based interface, it requires a calibration before operational use. The trigger for the calibration was upgraded in comparison to previous versions (Hussain et al., 2016; Franco et al., 2019). The physical switch was replaced by a copper pad that acts as a touch button. The calibration procedure is as follows: the touch button toggles the e-Cap from an operational status to a calibration status; once in the calibration status, the user perceives a vibration, and must raise the eyebrows to record the maximum value of the envelope of the sEMG signal; a threshold is automatically set as described in Franco et al. (2019).

### 2.3 3D-Printed Electrodes

The most critical part of the e-Cap is the electrode-skin interface. A lot of research exists in literature that deals with the problem of acquiring sEMG signals reliably (Day, 2002; Laferriere et al., 2011). Dry electrodes are preferred due to hygienic considerations and the advantage of being reusable, and are better suited for long-term measurements due to their more stable impedance compared to wet electrodes (Wolterink et al., 2020). We propose a solution which combines a reusable sticky plastic tape and non-gelled 3D-printed flexible TPU-based sEMG electrodes developed by the University of Twente (Wolterink et al., 2017). As shown in Figure 4 the sticky tape is transparent and allows easy and accurate electrode positioning. To increase wearability, the electrode interface was electrically and mechanically coupled to the e-Cap, by means of a magnetic connector.

### 2.4 Assistive Elbow Orthosis

**FIGURE 5:**
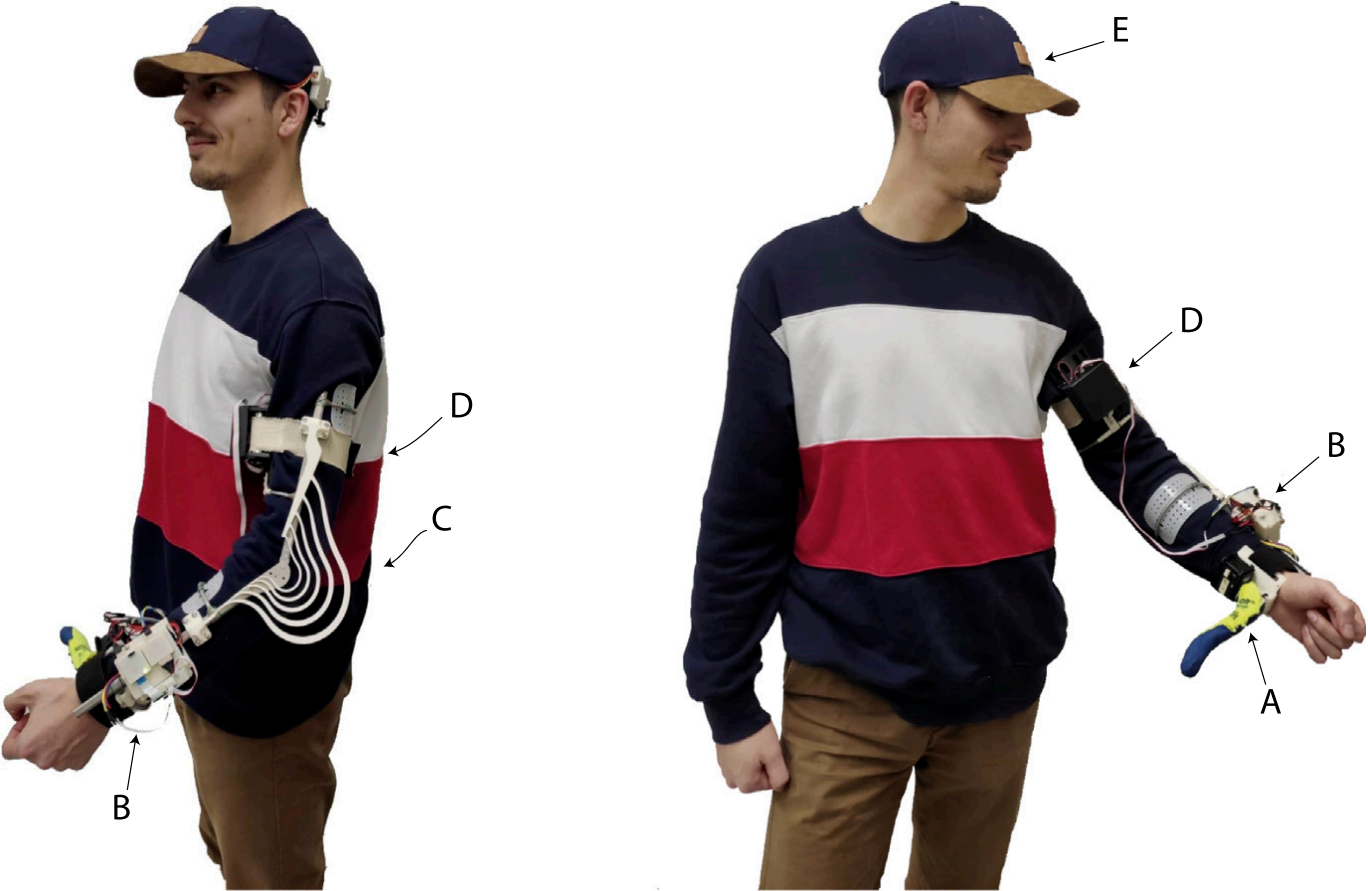
The Robotic sixth finger **(A)** and its power supply and control system **(B)**, developed by US, has been integrated with the Assistive Elbow Orthosis **(C)**, developed by UT, and the CUFF **(D)** from UP. The CUFF has been modified to be completely wearable. Motion of the finger is controlled *via* a new e-Cap version **(E)** developed by US with novel 3D-printed electrodes by UT.

**FIGURE 6:**
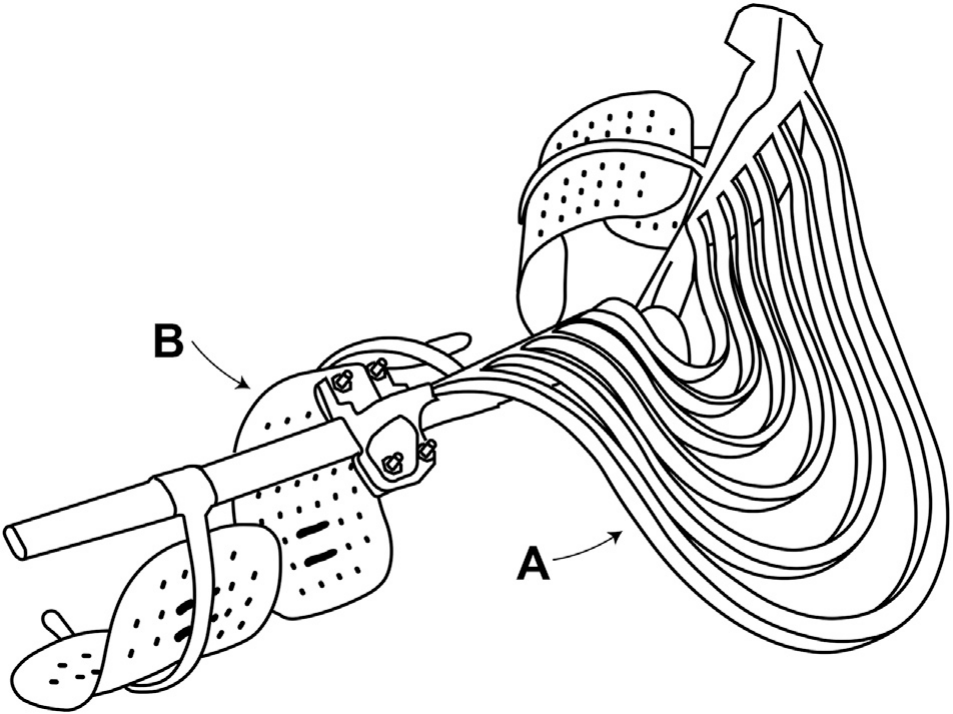
The Assistive Elbow Orthosis. **(A)** Lateral stack of 3D-printed nested gravity-balancing springs. **(B)** Metal brace with plastic supports [Wilmer elbow orthosis Plettenburg (2007)].

Tschiersky et al. (2019) have developed the Assistive Elbow Orthosis at the University of Twente as a wearable assistive device designed to aid in the lifting of the forearm by providing a gravity-balancing moment to the elbow joint of the wearer, see Figure 5. As shown in Figure 6, the device consists of a modified Wilmer elbow orthosis (Ambroise, Enschede, Netherlands) (Plettenburg, 2007) that acts as the mechanical interface to the wearer, and a stack of nested springs which is mounted laterally onto the orthosis. The spring shape has been optimized to provide an angle-dependent moment, which counteracts the moment caused by gravity acting on the forearm.

### 2.5 CUFF

**FIGURE 7:**
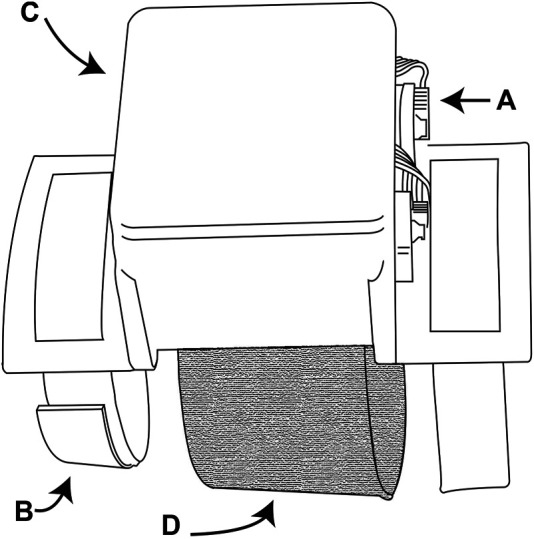
The CUFF. **(A)** Exposed electronics (motor encoders and connection plugs). **(B)** Velcro straps used to wear the device. **(C)** 3D-printed box enclosing the electronic board. **(D)** Tissue band attached to the motors for squeezing the arm.

The CUFF is a wearable haptic device that is able to provide both pressure and skin stretch information to the user’s arm. The device is composed of a structural frame, two mechanical actuation units and the feedback interface. Each actuation unit is powered by a Maxon DCX16S motor and equipped with a two-stage planetary gear-head with a gear ratio of 44:1. The maximum continuous power of each motor is 2.5 W. In Figure 7 a side view of the device is shown. The fabric band is attached to both motors, in such a way that, when actuated in a counter-rotating motion, the length of the tissue band is reduced, ultimately squeezing the arm. In the integrated device, the purpose of the CUFF is to provide force feedback proportional to the load on the Sixth Finger.

## 3 System Integration

In this section, we provide an explanation of how every component works within the system, and how we implemented hardware and software changes to build the device. In Figure 5 we show the setup worn and ready to be used. When the patient raises the eyebrows once, the e-Cap recognizes the gesture by sEMG real-time processing and triggers the Sixth Finger to close in order to perform a grasp.

This trigger signal is sent *via* a Bluetooth antenna to the Robotic Sixth Finger microcontroller, that starts closing itself until it reaches contact with an object. The contact sensing is implemented by setting a threshold on the Robotic Finger motor current. The input given by the patient is also acknowledged by the system, by providing a short vibration burst at the back of the head.

Once the Sixth Finger is in contact with the object, the patient can decide to increase the strength of the grasp by simply keeping the eyebrows raised. The amount of exerted force is proportional to the time the patient held the eyebrows up, and the value of the Sixth Finger current is sent in real-time to the CUFF device *via* the RS-485 interface. The value of the current is scaled to match the input range of the CUFF and then sent to the CUFF which renders the force feedback in real-time.

To extend the finger, the patient has to raise the eyebrows twice consecutively. This movement is acknowledged by the e-Cap by a double vibration on the occipital area of the user’s head. The integration process required modifications to all components. The Assistive Elbow Orthosis device is essential to help the stroke patients to use the system. As stated before, all devices are indirectly attached to the arm *via* the exoskeleton. To increase wearing comfort, two out of four plastic ergonomic supports have been removed and replaced by the Sixth Finger and the CUFF using custom 3D-printed mechanical interfaces (see Figure 5). Furthermore, the dimensions of the spring were adjusted to accommodate for the increased weight due to the added devices.

Customized code was implemented to map the sensed load of the Sixth Finger motor to the CUFF device. This task was challenging since the devices were developed independently from each other and the CUFF features a proprietary firmware, loaded onto its control unit. Moreover, the CUFF interface code, available at Malagia and Poggiani (2019), was written to control the device from a personal computer. In order to run on a microcontroller, the control library was modified to use an UART port instead of a USB port.

To use the UART port, additional hardware was necessary to allow for communication *via* the RS-485 protocol used by the CUFF device. To this end a MAX3485 chip was used to connect the Robotic Sixth Finger and CUFF communication pins. This was a suitable solution, since both devices use a 3.3 V power supply and support high data transmission rates, up to 10 Mbps. A small PCB was used to interpose the additional electronics between the Sixth Finger and the CUFF. With these modifications, the entire control library could be utilized to control the CUFF movements by the Sixth Finger microcontroller.

The CUFF was modified to increase wearability in the integrated system. To provide feedback the device has a fabric strip that goes around the arm. Patients, however, can have difficulties while wearing the CUFF, because the fabric strip can get stuck on the arm. Thus, the fabric strip has been modified by cutting it at the middle and sewing big velcro pads to both ends of the fabric. The CUFF is powered by the same 12 V battery pack as the Sixth Finger. A button was added to be used as an additional redundant control interface, since previously users reported problems using the e-Cap.

## 4 Pilot Studies

We have conducted a pilot study on the usability of the SoftPro Wearable System involving three patients (all male, average age 64.4). Two subjects taking part in the experiment were in acute phase (they have been affected by stroke no more than 3 months before the test) and one subject was in chronic state. The device can be used by subjects showing a residual mobility of the arm. For being included in the pilot experiment, patients had to score ≤2 when their motor function was tested according to the National Institute of Health Stroke Scale (NIHSS), item 5 “paretic arm.” Moreover, the patients showed the following characteristics: normal consciousness (NIHSS, item 1a, 1b, 1c = 0), absence of conjugate eyes deviation (NIHSS, item 2 = 0), absence of complete hemianopia (NIHSS, item 3 ≤ 1), absence of ataxia (NIHSS, item 7 = 0), absence of completely sensory loss (NIHSS, item 8 ≤ 1), absence of aphasia (NIHSS, item 9 = 0), absence of profound extinction and inattention (NIHSS, item 11 ≤ 1). Patients wore the system on the paretic upper limb, the left arm for two subjects and the right one for the other. Due to the design of the device, the same prototype can be worn on either the right or the left arm. Written informed consent was obtained from all participants. The procedures were in accordance with the Declaration of Helsinki.

**FIGURE 8:**
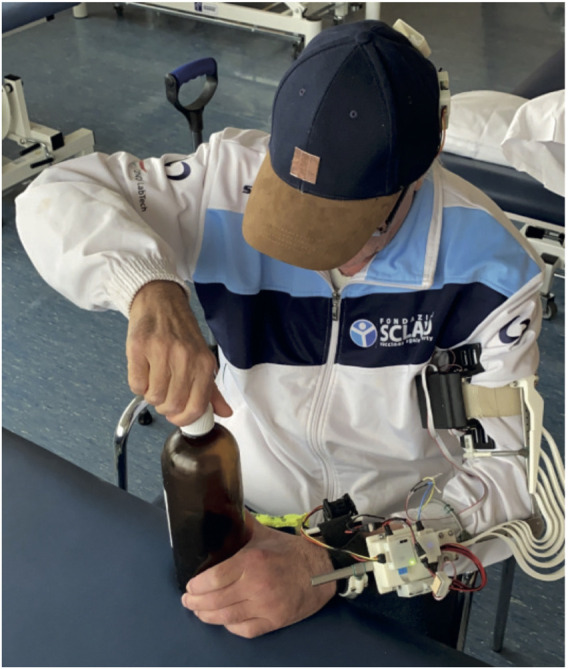
A stroke patient opening a bottle with the help of the SoftPro wearable system.

Patients were asked to wear the system, familiarize with the controller and then use the system to execute a series of bimanual tasks, representing common ADLs: opening a bottle (see Figure 8), removing the cap from a jar, and peeling an apple. After 30 min of use, we asked the participants to answer the ten questions of the system usability scale (SUS) (Brooke, 1996). The SUS is used to evaluate subjective assessments of usability. SUS yields a single number that represents a composite measure of the overall usability of the system being studied. It is a Likert scale where each item can be given a mark ranging from 1 “strongly disagree” to 5 “strongly agree.” SUS scores range from 0 to 100, where 0 means “awful” and 100 represents “excellent.” A SUS score above a 68 is considered above average. Details on how to compute the final mark can be found in Brooke (1996).

## 5 Results

The obtained SUS scores are 70, 95, and 90. This means that the system was deemed useful and very easy to use. We also collected some suggestions for further improvements of the system. One patient suggested to provide the ability of adapting the finger length depending on the task. Two patients perceived the haptic feedback from the CUFF as not very useful during operations and suggested to reduce the bulkiness of the feedback device. They also suggested to limit the force feedback to the grasping phase and remove it once a stable grasp is achieved. One patient preferred the push button for control and stated that a cap could not be comfortably used indoors.

Throughout all experiments, the Assistive Elbow Orthosis was instrumental in allowing the patients to complete their tasks. Even though they criticized the bulkiness of the 3D-printed springs, patients always struggled raising the paretic forearm with the Sixth Finger when not wearing the orthosis. Finally one patient suggested to add the ability of regulating the closing velocity and applied force through knobs embedded in the control box at the forearm.

## 6 Discussion

In this paper, we successfully integrated different devices to help people keep using their impaired upper limb. Patients with reduced mobility of the hand often stop moving the affected limb, loosing the muscular tone recovered during the rehabilitation period. The compensation offered by using the SoftPro system motivates the patient to use her or his muscles by encouraging the patients to use their residual abilities effectively, instead of being solely dependent on the motion of a robotic device. The advantage of the proposed system compared with the constraint-induced movement therapy—a rehabilitative approach characterized by the restrain of the healthy upper limb accompanied by the shaping and repetitive task-oriented training of more affected upper extremity, with the purposes of overcoming the learned nonuse phenomenon of the hemiplegic upper extremity (Taub et al., 1993)—is that there is no need to immobilize or restrain the healthy limb to encourage the use of the paretic hand.

The final outcome of the study showed that, although the device had a very positive effect on patients who reported that they would like to use it in daily life, some crucial points need to be reviewed and evaluated.

One unanswered question concerns the actual need for such a complex system vs. a more simple solution, e. g., the Robotic Sixth Finger without any other device connected. We should evaluate whether patients would prefer to use only the Sixth Finger due to the lower encumbrance, or if it is preferable to use the entire system which offers more arm mobility and more fine control, including feedback. All 3 patients who tested the system reported that, although the functionality of the device helped to move and use the upper limb, its size may reduce its usability. An aspect to be investigated in future studies is the usability of the system in domestic contexts with different usage conditions, e.g., sitting and standing.

The control interface is another crucial aspect to be considered for assistive devices designed for long-term use. The subjects who used our system reported that they were interested in testing different input devices for the integrated system, since they were not always comfortable using the e-Cap. Previous studies reported that some patients prefer to use the e-Cap because they perceive the interface at the frontalis muscle as very intuitive (Salvietti et al., 2017), whereas some other patients prefer a button to trigger the opening and closing of the Sixth Finger, together a knob to control its grasp strength. In the future, the 3D-printed sEMG electrodes described in Section 2.3 could be integrated into the e-Cap and be printed in one go. Furthermore, 3D-printing potentially allows for easy adaptations, improving customizability.

The pilot study involving patients also offered the opportunity to collect important suggestions from potential final users that can guide the future development of the device. For instance, one patient asked for a solution to turn the pages of a book, since this activity of daily living is very important to him. His suggestion was to reduce the length of the Sixth Finger, which led us to the conclusion that adding a length adjustment capability to Sixth Finger could greatly expand its possible uses. Finally, all patients underlined the need for customization and reduction of encumbrance. To this end, the Assistive Elbow Orthosis could be modified to reduce the size of the spring and the CUFF could also be reduced in size and placed at the forearm to make the design more compact.

## 7 Conclusion

The aim of this study is to present a novel device for grasping compensation in motor-impaired subjects. The SoftPro Wearable System is the result of the integration of different technologies, such as a supernumerary robotic finger, an sEMG input device using 3D-printed electrodes, a haptic feedback device and a gravity-balancing arm orthosis.

A pilot study was conducted involving three stroke patients. The positive feedback from the subjects confirmed the need for technological advances and novel concepts in the field of assistive devices. The first impressions of the users, collected by the authors, will serve as guidance for subsequent development of portable assistive devices. The proposed technical solution could also be used by spinal cord injury patients, considering that this category is also subject to upper-limb paresis.

## Data Availability

The original contributions presented in the study are included in the article/Supplementary Material, further inquiries can be directed to the corresponding author.

## Footnotes

1
https://www.softpro.eu
